# The Use of the Ambulatory Arterial Stiffness Index in Patients Suspected of Secondary Hypertension

**DOI:** 10.3389/fcvm.2016.00050

**Published:** 2016-12-15

**Authors:** Joshua R. A. Verbakel, Ahmet Adiyaman, Nicole Kraayvanger, Dirk G. Dechering, Cornelis T. Postma

**Affiliations:** ^1^Division of Vascular Medicine, Department of Internal Medicine, Radboud University Nijmegen Medical Centre, Nijmegen, Netherlands; ^2^Department of Cardiology, Isala Hospital, Zwolle, Netherlands; ^3^Rijnstate Hospital, Arnhem, Netherlands; ^4^Department of Cardiovascular Medicine, University Hospital of Münster, Münster, Germany

**Keywords:** hypertension, renal artery, atherosclerosis, arterial stiffness, diagnosis, differential, difficult-to-treat hypertension, secondary hypertension

## Abstract

The ambulatory arterial stiffness index (AASI) is a marker of arterial stiffness and is derived from ambulatory 24-h blood pressure registration. We studied whether the AASI could be used as a predictive factor for the presence of renal artery stenosis (RAS) in patients with a suspicion of secondary hypertension and as such as a diagnostic tool for RAS. We included 169 patients with difficult-to-treat hypertension. They all underwent 24-h ambulatory blood pressure monitoring registration, imaging of the renal arteries, and cardiovascular risk measurement, including smoking, history, biometrics, blood pressure, renal function, lipids, and glucose metabolism. Performing univariate and multivariate analyses, we investigated if AASI and the other cardiovascular risk factors were related to the presence of RAS. Of the 169 patients (49% women), 31% had RAS. The mean AASI was 0.44 (0.16). The presence of RAS showed no significant correlation with AASI (*r* = 0.14, *P* = 0.06). Age (*r* = 0.19, *P* = 0.01), hypercholesterolemia (*r* = 0.26, *P* = 0.001), history of CVD (*r* = 0.22, *P* = 0.004), and creatinine clearance (*r* = −0.34, *P* < 0.001) all demonstrated a correlation with RAS. Although AASI is higher in patients with RAS, AASI does not independently predict the presence of RAS in hypertensive subjects.

## Introduction

Increased arterial stiffness results from reduced elasticity of the arterial wall and is an independent predictor for cardiovascular risk. The ambulatory arterial stiffness index (AASI) is an indirect arterial stiffness index, which can be simply calculated from 24-h ambulatory blood pressure monitoring (ABPM) and has proven to be associated with cardiovascular adverse events and vascular damage, over and beyond 24-h pulse pressure and mean arterial pressure (1–3).

Hypertension is one of the major causes in the development of atherosclerotic vascular and organ damage and decline in renal function. Increasing arterial stiffness is a forerunner and early sign of atherosclerotic disease and is associated with and a consequence of hypertension (4). Atherosclerotic renal artery stenosis (RAS) contributes to the development of hypertension, but atherosclerotic RAS in itself is also an important contributing factor in the development of atherosclerotic complications apart from the accompanying hypertension (5).

Therefore, it can be hypothesized that arterial stiffness might be higher in the case of concomitant hypertension and RAS than in the case of hypertension without RAS. If this proves to be so, it could be used to distinguish patients with RAS from those without it. The arterial stiffness index could possibly aid in the detection and diagnosis of RAS. In treating hypertensive patients, it can have implications to know whether or not a renal arterial obstruction is present, and it might be valuable to look for this condition, therefore, particularly in cases of hypertension that are difficult to treat. In such cases, it is advisable to choose the least invasive screening option. As the AASI is derived from an ABPM that can be obtained without too much inconvenience to patients, it could be a real step forward if the AASI could be used in this respect.

Therefore, we studied whether the AASI is associated with the presence of RAS in patients with hypertension and the clinical suspicion of secondary hypertension, more in particular of the presence of atherosclerotic RAS. RAS caused by fibromuscular dysplasia (FMD) is much less common, has a distinct pathophysiology, and is not a subject of this study.

## Patients and Methods

We included 169 consecutive patients with a clinical suspicion of secondary hypertension mainly based on difficult-to-treat hypertension indicated by a persistent blood pressure over 140/90 mmHg, who were referred to our tertiary hypertension unit. The study was retrospective in nature, and we used a pre-specified protocol. All included patients underwent imaging of the renal arteries and 24-h ABPM. Patients were excluded if they were diagnosed with a parenchymal renal disease other than that caused by atherosclerotic or hypertensive changes. Subjects with diabetic nephropathy with proteinuria >1.0 g/day were also excluded.

### Ethical Considerations

This study was retrospective in nature and had no effect on the treatment of patients who enrolled. No experimental investigations were done, and all data were obtained in the course of usual patient care management. The data were completely de-identified. According to the guidelines of the institutional ethics board, it was not necessary, therefore, to obtain written informed consent of all patients. All conditions of the latest version of the Declaration of Helsinki of the World Medical Association were met.

### Measurement of RAS

State-of-the-art transcatheter angiographies [intra-arterial (i.a.) TCA], CT angiographies (CTA), or magnetic resonance angiographies (MRA) of the renal arteries were made in all patients. The state-of-the-art MRA and CTA result in reliable estimates of renal artery obstruction in comparison to i.a. TCA (6). If there was a high suspicion of FMD, primarily an i.a. TCA was done. If there was any doubt in interpreting the lesions found on CTA or MRA, an i.a TCA was made.

Each renal artery was analyzed for the presence of stenosis, which was graded on the basis of the most severe reduction of arterial diameter compared with an uninvolved renal artery segment proximal or distal to the stenosis. A renal artery was graded as normal if no luminal obstruction was present. A stenosis was considered high-grade if it was over 50% of luminal surface and low-grade if it was under 50% of luminal surface.

According to the radiological reports, which were conducted by radiologists experienced in this field, subjects were classified into three groups as (1) having no RAS, (2) having low-grade RAS (<50%), and (3) having at least one high-grade RAS (>50%). Baseline and follow-up clinical and biochemical data were gathered through a pre-specified protocol. The angiographies and the ABPM measurements were done during the initial work-up of the patients after referral to our institution.

### Blood Pressure Measurements

Hypertension was defined according to European guidelines as conventional systolic blood pressure (SBP) ≥140 mmHg and/or diastolic blood pressure (DBP) ≥90 mmHg. Experienced clinicians measured conventional blood pressure by computing the mean of three blood pressure recordings after resting 10 min in the semi-supine position, with the cuff placed at the level of the heart and patients’ legs uncrossed. ABPM recordings were programed at 15–30-min intervals during daytime and at 30–60-min intervals during nighttime. ABPM quality criteria included an interval range of 15 min, with at least five readings between midnight and 0600 hours and at least 15 daytime readings (7). If these criteria were not met, patients were excluded. The validated monitors used, were the oscillometric SpaceLabs 90207 (SpaceLabs Inc., Redmond, Washington, DC, USA), the Oxford Medilog (Oxford Medical Systems Ltd., Oxford, UK), and the oscillometric Mobil-O-Graph (I.E.M., Stolberg, Germany).

The regression slope of diastolic on SBP was computed from the 24-h ABPM recordings. AASI was defined as one minus the regression slope (8). We used the coefficient of determination (r^2^) as a measure of goodness of fit of the AASI regression line (9).

### Other Measurements

We used the questionnaires originally administered to obtain information on each subject’s medical history and smoking and drinking habits. The following data were also retrieved: age, sex, height, weight, body mass index (BMI, the weight in kilograms divided by the square of the height in meters), duration of hypertension, and the number of antihypertensive drugs. Hypertensive patients received antihypertensive drugs with a diastolic target BP ≤90 mmHg and systolic <140 mmHg or lower according to comorbid conditions (10).

Renal function was measured by estimating creatinine clearance, using the Cockroft–Gault formula (11). Renal failure was defined as proteinuria (defined as urinary protein in concentrations greater than 0.3 g in a 24-h urine collection) or a glomerular filtration rate of less than 90 mL/min/1.73 m^2^. End-stage renal failure was defined as a glomerular filtration rate of less than 15 mL/min/1.73 m^2^.

We measured serum cholesterol and blood glucose by automated enzymatic methods. Hypercholesterolemia was defined as a serum total cholesterol level of ≥5.2 mmol/L or the intake of lipid-lowering drugs. Diabetes mellitus type was a self-reported diagnosis, or a fasting or random blood glucose level of at least 7.0 mmol/L (126 mg/dL) or 11.1 mmol/L (200 mg/dL), respectively, or the use of anti-diabetic drugs. Presence of cardiovascular disease (CVD) was defined as a medical history with coronary artery disease (defined as ischemic changes on exercise tolerance testing or by significant obstruction on coronary angiography), myocardial infarction, transient ischemic attack, ischemic stroke or death from any cardiovascular cause, peripheral arterial disease, or vascular surgery.

## Statistical Analysis

For statistical analysis and database management, we used SPSS v.22 for Windows (SPSS Inc., Chicago, IL, USA). We compared means and proportions by ANOVA and the χ^2^ statistic, respectively, and evaluated unadjusted associations with Pearson’s correlation coefficient. To evaluate the association between AASI and RAS, we calculated unadjusted, age- and sex-adjusted and multivariable-adjusted odds ratios (ORs) plus 95% confidence intervals (CIs), with the study subgroup without RAS set as the reference group. In regression analysis, we adjusted for selected baseline characteristics that are known to increase risk of RAS, including sex, age, BMI, current smoking, diabetes mellitus, hypercholesterolemia, previous CVD, and family history of CVD.

## Results

### Patient Characteristics

The 169 participants included 83 females (49.1%). Blood-pressure-lowering drugs at the time of referral were used by 154 (91.1%) of the patients. This medication had been stopped in a few patients because of insufficient effect before referral. The number of different antihypertensive medications per patient was 2.3 ± 1.3. Mean (±SD) age was 48.0 ± 13.5 years.

At enrollment, 66 participants (39.1%) were current smokers; 61 (36.1%) reported intake of alcohol; 89 (52.7%) reported a history of CVD; 54 (32.0%) showed hypercholesterolemia or were treated for it; 25 (14.8%) had diabetes mellitus; and 14 (8.3%) had renal insufficiency, 1 of whom had end-stage renal failure.

In the whole study population, the mean (±SD) 24-h blood pressure averaged 152.4 ± 20.0 mmHg SBP and 94.3 ± 13.8 mmHg DBP. The 24-h mean (±SD) arterial pressure averaged 114.8 ± 15.0 mmHg. The mean heart rate was 73.1 ± 13.6 bpm. The mean (±SD) AASI was 0.44 ± 0.16.

An i.a. TCA of the renal arteries was obtained in 129, a CTA in 21, and an MRA in 29 patients. RAS was diagnosed in 52 participants (30.8%), 32 (18.9%) of whom had severe RAS with a luminal obstruction over 50%, and 7 (4.1%) of the entire group with a stenosis had bilateral RAS over 50%. Table 1 lists the main clinical characteristics of subjects with and without RAS.

**Table 1:** **Baseline characteristics of participants categorized by renal artery stenosis (RAS)**.

	All	No RAS	RAS	*P-*value
				RAS versus no RAS
Number of patients (% women)	169 (49)	117 (50)	52 (46)	0.61
Age (years)	48.0 (±13.5)	46.3 (±13.5)	51.8 (±12.8)	0.01
BMI (kg/m^2^)	27.4 (±5.0)	27.8 (±5.1)	26.6 (±4.9)	0.17
Height (cm)	170.3 (±9.8)	171.0 (±10.5)	168.8 (±9.0)	0.18
Smoking (%)	66 (40)	41 (35)	25 (38)	0.12
Hypercholesterolemia (%)	54 (32)	28 (24)	26 (48)	0.001
LDL cholesterol (mmol/L)	3.3 (±1.1)	3.2 (±1.0)	3.6 (±1.3)	0.10
Non-HDL cholesterol (mmol/L)	4,0 (±1.2)	4,7 (±2.2)	4,2 (±1.5)	0.02
Fasting blood glucose (mmol/L)	5.7 (±1.8)	5.7 (±1.9)	5.7 (±1.6)	0.92
Cardiovascular disorder (%)	89 (53)	53 (45)	36 (69)	0.004
Renal failure (%)	14 (8)	8 (7)	6 (12)	0.31
Creatinine clearance (mL/min)	101.4 (±32.8)	108.7 (±32.2)	84.9 (±28.3)	<0.001
Number of antihypertensive drugs	2.3 (±1.3)	2.4 (±1.3)	2.1 (±1.3)	0.29
**24-h ambulatory measurements**				
Systolic pressure (mmHg)	152.4 (±20.0)	151.6 (±18.7)	154.2 (±22.9)	0.44
Diastolic pressure (mmHg)	94.3 (±13.8)	94.8 (±13.7)	93.1 (±13.8)	0.45
Mean arterial pressure (mmHg)	114.8 (±15.0)	114.6 (±14.4)	115.0 (±16.4)	0.87
Heart rate (bpm)	73.1 (±13.6)	73.4 (±12.6)	72.2 (±15.8)	0.62
AASI	0.44 (±0.16)	0.43 (±0.15)	0.48 (±0.17)	0.06

*Data are given in mean numbers unless indicated otherwise; ± indicates one SD, % indicates percentage of that group*.

*RAS, renal artery stenosis; BMI, body mass index; LDL, low density lipoprotein; HDL, high density lipoprotein; AASI, ambulatory arterial stiffness index; bpm, beats per minute*.

*Creatinine clearance was calculated by the Cockroft–Gault formula*.

### Unadjusted Analysis

**Figure 1:**
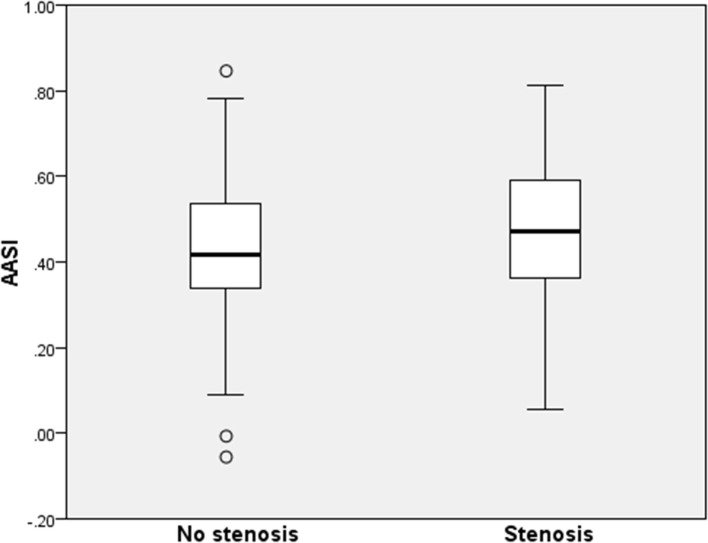
**Ambulatory arterial stiffness index stratified for the presence of renal artery stenosis (RAS)**. A boxplot is shown with median and upper and lower quartiles. The *P*-value between groups is 0.06. No stenosis indicates the patients with no RAS. Stenosis indicates the patients without a RAS.

Both groups included nearly 50% women. Subjects with RAS were older (51.8 versus 46.3, *P* = 0.01), had a marginally lower BMI (26.6 versus 27.8 kg/m^2^, *P* = 0.17), and more often had hypercholesterolemia (48 versus 24%, *P* < 0.001), a history of CVD (69 versus 45%, *P* = 0.004) or renal failure (12 versus 7%, *P* = 0.31) with an average creatinine clearance of 84.9 versus 108.7 mL/min (*P* < 0.001). The AASI in patients with RAS versus without RAS was 0.48 ± 0.15 (CI ±0.03 range 0.40–0.46) versus 0.43 ± 0.17 (CI 0.05 range 0.43–0.53) (*P* = 0.06) (Figure 1). In the RAS group, the seven patients with a bilateral significant RAS had the highest mean AASI of 0.56. Further separate analysis of this small group is not meaningful.

### Multivariable Analysis

In all 52 subjects, RAS was independently associated with creatinine clearance with an OR of 20.49 (*P* < 0.001) (Table 2). RAS was not independently associated with AASI, age and history of CVD, whereas the association with non-HDL cholesterol was borderline significant (OR 3.53, *P* = 0.06).

**Table 2:** **Univariable and multivariable odds ratio (OR) models for presence of renal artery stenosis (RAS) in 52 hypertensive patients with RAS**.

	Univariable OR	95% confidence intervals (CI)	*P*-value	Multivariable OR	95% CI	*P*-value
Male gender	1.19	0.62–2.28	NS			
Age	1.03	1.01–1.06	0.02	0.15	0.96–1.03	0.70
Bodymass index	0.95	0.89–1.02	NS			
Height	0.09	0.00–3.07	NS			
Smoking	1.69	0.87–3.29	NS			
Hypercholesterolemia	3.18	1.60–6.34	0.001			
LDL cholesterol	1.34	0.94–1.90	NS			
Non-HDL cholesterol	1.33	1.01–1.77	0.05	3.53	0.99–1.56	0.06
Fasting blood glucose	1.01	0.83–1.23	NS			
Cardiovascular disorder	2.72	1.36–5.43	0.05	2.04	0.85–2.83	0.15
Renal failure	1.78	0.58–5.41	NS			
Creatinine clearance	0.97	0.96–0.99	<0.001	20.49	0.96–0.99	<0.001
Number of antihypertensive drugs	0.87	0.67–1.12	NS			
**24-h ambulatory measurements**						
Systolic pressure	1.01	0.99–1.02	NS			

## Discussion

In this study population, the AASI was not independently associated with the presence of RAS in patients with a suspicion of secondary hypertension. In the univariate analysis, higher age, lower creatinine clearance, hypercholesterolemia, and history of CVD were associated with RAS, whereas only lower creatinine clearance was independently associated with RAS.

Renal artery stenosis carries a high risk for cardiovascular complications and is associated with increasing mortality. Even low-grade RAS, defined as RAS <50% of the arterial lumen, is associated with future cardiovascular events (12). Therefore, it is important to be able to identify patients suffering from this condition. In the search for secondary hypertension, the diagnostic evaluation of hypertensive patients is challenging because multiple requirements have to be fulfilled. Ideally, a screening technique should be accurate, reliable, non-invasive, and not prone to complications, as is the case with nephrotoxic contrast agents necessary in the definitive diagnosis of RAS. Before turning to invasive investigations, it would be very useful to identify the patients with the greatest risk of this condition, so that the number of invasive procedures could be limited.

There are at present several specific clinical and biochemical clues that can suggest various forms of secondary hypertension (13). If present, these can guide investigations that are supplementary to the routine screening tests. In addition, various clinical risk factors can be identified that point to secondary hypertension in general without specific clues as to what kind of secondary hypertension might be involved (14). These include poor response to adequate therapy, worsening of control in previously stable blood pressure, age of onset younger than 20 or older than 50, and various others (14). It is important to identify secondary hypertension because cardiovascular morbidity and mortality are determined to a large extent by clinical conditions that involve the arterial system.

The presence of atherosclerotic vascular lesions is in itself also a very strong indicator of cardiovascular morbidity and mortality (15). The presence of an atherosclerotic RAS, even a less than 50% stenosis, is accompanied by a high risk of cardiovascular complications as compared to hypertension without RAS (8, 13, 16–18). A cohort of patients with RAS, detected at the time of coronary angiography, showed a 4-year survival rate of 65% as opposed to 86% for those without RAS (16). Most patients with RAS also have renal insufficiency in varying severity and diminished renal function in general. A decline in renal function is also an important independent contributing factor to the development of CVD complications (19).

One expression of vascular damage is increased arterial stiffness, which as such is an important marker and predictor of CVD complications (15, 16). This parameter is also recognized as a marker of subclinical target organ damage.

Taking these corroborations together, it seemed likely that hypertensive patients with RAS would show greater arterial stiffness when compared to those without RAS and that the difference might be such that it might be possible to distinguish between these groups based on arterial stiffness. We chose the AASI as the measure of arterial stiffness because this information is readily available from 24-h ABPM, which is regarded as the golden standard of blood pressure measurement. Several studies confirmed the clinical usefulness of the AASI as a measure of arterial stiffness as well as its prognostic significance for the appearance of cardiovascular complications in patients with hypertension (1–3, 9, 19, 20).

In the present study, AASI did show an independent relation with RAS but only to bilateral RAS and not to unilateral stenosis. However, the number of patients with bilateral RAS is too small to permit definitive conclusions. Could the number of patients overall in this study be too small to detect a significant difference? Given the CI of the means and the differences between the two groups, it is doubtful whether such a difference is in fact present. From the number of patients in this study and the outcomes, a power of 0.5 could be calculated. To get a power of 0.8, the number of patients in each group would have to be 163.

On the basis of these results of this study, it seems reasonable to conclude that there is no clinically important difference in AASI between these two groups. Almost all patients were on hypertensive medication when the ABPM values were obtained. A recent study showed convincingly that antihypertensive treatment has only a marginal effect on the AASI over time (21). So, the use of medication has certainly not had enough effect to distort the results of this study. AASI showed a correlation with age, systolic 24-h blood pressure, and 24-h heart rate. There was also an independent relation between RAS and higher age, higher BMI, raised LDL cholesterol level, lowered GFR, and a history of CVD. These are all well-known indicators of severe vascular damage and heralds of further CVD complications.

So, the hypertensive patients in this study showed the confirmed parameters of advanced vascular disease, but the AASI was not one of them, only among those with bilateral stenosis, a group known for its high risk of imminent CVD complications (22). The AASI and, hence, arterial stiffness did not show an independent relation with RAS. This seems contrary to expectations as the relation between arterial stiffness and CVD complications is so strong (1–3, 9, 19, 20, 23–26).

There are some possible explanations for this. First, the average AASI in the group without RAS was 0.43; the average AASI in the group with RAS was 0.48; and in the group with bilateral RAS it was 0.56. These values are in the highest quintiles, as was shown in a study of hypertensive patients (9). Those with an AASI in these upper quintiles had the highest risk of CVD complications (9). The AASI as an indirect measure of vascular stiffness in patients with hypertension with or without a RAS in this study, therefore, are already in the highest proportion of this measure. As all groups are at an advanced stage of vascular damage with a high degree of arterial stiffness, a further significant difference between these patients may not be identifiable.

This study was not aimed at directly evaluating whether AASI correlates with arterial stiffness, as we performed no direct measure of arterial stiffness such as pulse wave velocity. We merely studied the relation of an indirect measure of arterial stiffness with the presence of a serious atherosclerotic complication in hypertensive patients. Another matter could be the generalizability of the outcome. The patients in this study had difficult-to-treat hypertension and other clinical or biochemical indicators suggesting secondary hypertension. Since there were no other selection criteria for this specific group, they can be considered representative for this group of hypertensive patients in general (24).

Though we did indeed reveal that hypertensive patients with RAS have a mean higher AASI than those without, this difference was not large enough to be useful as a distinctive feature. So, in this study, we could not find a value of the AASI that indicates the presence of RAS. Therefore, we could not advise introducing the AASI in the clinical decision-making in hypertensive patients suspected of secondary hypertension.

## Author Contributions

All authors listed, have made substantial, direct, and intellectual contribution to the work, and approved it for publication.

## Conflict of Interest Statement

The authors declare that the research was conducted in the absence of any commercial or financial relationships that could be construed as a potential conflict of interest.
